# Combined Impacts of Acute Heat Stress on the Histology, Antioxidant Activity, Immunity, and Intestinal Microbiota of Wild Female Burbot (*Lota Lota*) in Winter: New Insights into Heat Sensitivity in Extremely Hardy Fish

**DOI:** 10.3390/antiox14080947

**Published:** 2025-07-31

**Authors:** Cunhua Zhai, Yutao Li, Ruoyu Wang, Haoxiang Han, Ying Zhang, Bo Ma

**Affiliations:** 1 Heilongjiang River Fishery Research Institute, Chinese Academy of Fishery Sciences, Harbin 150070, China; 2 National Agricultural Experimental Station for Fishery Resources and Environment, Fuyuan, Ministry of Agriculture, Harbin 150070, China; 3 Key Laboratory of Cold Water Fish Germplasm Resources and Multiplication and Cultivation of Heilongjiang Province, Heilongjiang River Fishery Research Institute, Chinese Academy of Fishery Sciences, Harbin 150070, China

**Keywords:** *lota lota*, heat stress, antioxidant, immune, apoptosis, microbiomics

## Abstract

Temperature fluctuations caused by climate change and global warming pose a threat to fish. The burbot (*lota lota*) population is particularly sensitive to increased water temperature, but the systematic impacts of high-temperature exposure on their liver and intestinal health remain unclear. In January of 2025, we collected wild adult burbot individuals from the Ussuri River (water temperature: about 2 °C), China. The burbot were exposed to 2 °C, 7 °C, 12 °C, 17 °C, and 22 °C environments for 96 h; then, the liver and intestinal contents were subsequently collected for histopathology observation, immunohistochemistry, biochemical index assessment, and transcriptome/16S rDNA sequencing analysis. There was obvious liver damage including hepatocyte necrosis, fat vacuoles, and cellular peripheral nuclei. Superoxide dismutase (SOD), catalase (CAT), and glutathione peroxidase (GSH-Px) activities were elevated and subsequently decreased. Additionally, the malondialdehyde (MDA) level significantly increased with increasing temperature. These results indicate that 7 °C (heat stress temperature), 12 °C (tipping point for normal physiological metabolism status), 17 °C (tipping point for individual deaths), and 22 °C (thermal limit) are critical temperatures in terms of the physiological response of burbot during their breeding period. In the hepatic transcriptome profiling, 6538 differentially expressed genes (DEGs) were identified, while KEGG enrichment analysis showed that high-temperature stress could affect normal liver function by regulating energy metabolism, immune, and apoptosis-related pathways. Microbiomics also revealed that acute heat stress could change the intestinal microbe community structure. Additionally, correlation analysis suggested potential regulatory relationships between intestinal microbe taxa and immune/apoptosis-related DEGs in the liver. This study revealed the potential impact of environmental water temperature changes in cold habitats in winter on the physiological adaptability of burbot during the breeding period and provides new insights for the ecological protection of burbot in the context of global climate change and habitat warming.

## 1. Introduction

The global atmospheric temperature has increased by 1.80–4.01 °C over the past 100 years [1], posing a major threat to animals, especially cold-water fish. Studies have shown that many cold-water fish species are vulnerable to ambient temperature elevation. For example, heat stress can disrupt the homeostasis of the gut microbiota and decrease immunity and resistance to disease in rainbow trout (*Oncorhynchus mykiss*) [2,3] and enhance energy demands and mortality in Atlantic cod (*Gadus morhua*) [4]. In addition, exposure to 20 °C heat stress can affect the epithelial barriers of Atlantic salmon (*Salmo salar*) [5]. Cold-water fish may adapt internal regulatory mechanisms under high-temperature-stress conditions. However, there is limited evidence on the impacts of rising temperatures on the physiological response in burbot (*lota lota*).

Burbot is the only cold stenothermal member of the Gadidae family; it usually selects warmer temperatures (10–12 °C) for survival during non-reproductive periods [6]. In contrast, a normal spawning process and high percentage of hatched larvae have been observed at 2 °C (often under ice) [7,8]. Therefore, the physiological functions of this cold-water fish have a narrow temperature range. Hence, the elevation in river temperatures, due to factors such as impoundment, hydropower, flood management, and the global climate, can result in habitat losses and have negative effects on the spawning and embryo development of hatchery burbot [8,9]. Many burbot populations in Eurasia and North America have experienced declines or extirpation due to global warming [10]. In Bulgaria, burbot has been classified as endangered due to warming water temperatures in the Danube river [11]. In China, burbot is distributed in the waters of Northeastern China, such as the Ussuri, Songhua, Mudan, Nenjiang, and Erguna Rivers; burbot populations are also threatened in China and have been added to the list of Rare and Rare Aquatic Wildlife. Furthermore, the requirement for a colder habitat environment during the reproductive period highlights the potential vulnerability to increased temperatures in winter. Given the sensitivity of ectotherms to dynamic temperatures, a thorough understanding of the physiological response process to thermal stress in cold stenothermal fish is essential for fish ecology [6].

The intestine has broad interactions with other organs and is involved in regulating many physiological functions in the host [12]. The intestinal microbe community contributes to nutrient supplementation and is involved in the regulation of metabolism and the immune response [13,14]. Previous evidence has shown that temperature changes can induce the disruption of the intestinal microbiota community, which affects the welfare of aquatic animals [15]. However, the effects of heat stress on the gut microbiota of *lota lota* remain unclear.

The liver is a multifunctional organ that plays a pivotal role in metabolism, the immune system, and nutrient absorption in crustaceans [16]. Meanwhile, the liver is also thought to crosstalk with the intestinal microenvironment [17]. The gut–liver axis plays a critical role in maintaining physiological homeostasis in vertebrates [18]. Microbe metabolites and endotoxins may be transported to the liver via the portal vein, which consequently affects normal hepatic functions, disrupting the conditions for the colonization of gut microorganisms [19,20]. Many studies have shown that various environmental pollutants affect the interactions of the microbe–gut–liver axis [21,22]. However, few studies have focused on whether the external environment plays a regulatory role in gut–liver communication.

The first aim of the present study was to provide a description of the responses to heat challenge in laboratory conditions, which were representative of field conditions, in an effort to understand the underlying physiological responses. To this end, we exposed burbot to thermal conditions. We measured key oxidative stress markers and observed histopathological damage in liver tissues following a 96 h thermal challenge. The second aim was to elucidate the molecular mechanisms of the dynamic effects of the thermal limit for burbot on the microbe–gut–liver axis during the reproductive period. To this end, 16S ribosomal DNA (16S rDNA) sequencing was performed to analyze the changes in intestinal bacteria taxa, and transcriptome analysis was carried out to determine the functional genes and relevant pathways in response to thermal stress. Our results provide theoretical support for conservation strategies for wild *lota lota* populations in the context of global warming.

## 2. Material and Methods

### 2.1. Animals Breeding and Sample Collection

The animal experiments in this research project were approved by the guidelines of the Laboratory Animal Ethics Committee of the Research Institute of Fisheries of the Heilongjiang River (No. 20241015-001).

**Figure 1:**
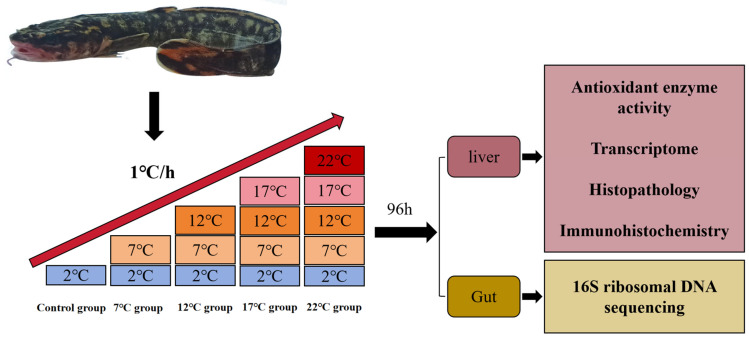
Experimental specimens and schematic diagram for the study design.

All experimental fish were sourced from the Ussuri River (China) using angling and mist-nets in January 2025 (water temperature: 2 °C) and were identified as female individuals based on the morphological classification method. In addition, roe from all subject individuals had reached stage IV of development maturity, indicating that the fish were in their reproductive period. All the fish were then transported to the indoor culture facility, and the external temperature was maintained at 1.8–2.3 °C. Prior to the formal experiment, all fish were habituated for 48 h to recover from transport stress in a temperature-controlled recirculating water tank (80.5 cm × 48 cm × 39 cm). The tap water was aerated for 6 h and replaced twice daily, the water temperature was maintained at 2 °C (proper temperature in the spawning period), and the pH varied from 6.5 to 7.5. In addition, dissolved oxygen (DO) remained between 6.0 and 7.8 mg/L, and the light cycle was 12 h light/12 h dark. A total of 150 healthy adult burbot (body length: 37 ± 2.5 cm; body weight: 307.48 ± 46.5 g) were randomly selected and assigned to five groups; each group contained three replicates with 10 individuals per tank. After all specimens were acclimated at 2 °C, a moderate heating scheme was applied by raising the water temperature at a relatively constant rate of 1 °C per hour until the water temperature reached 7 °C, 12 °C, 17 °C, and 22 °C (Figure 1). The experimental environmental conditions were the same as during the acclimatization period. During the experiment, the number of dead fish in each group at 96 h was recorded, and dead fish were removed promptly. After 96 h of treatment, 9 burbot from each group were randomly chosen and anesthetized using 0.02% tricaine methanesulfonate (MS-222; Sigma, Kalamazoo, MI, USA). Additionally, 300 mg of liver tissue was dissected and then homogenized with a cold extraction solution from the detection kits at a ratio of 1:9 (*w*:*v*). After centrifugation at 12,000 rpm for 10 min at 4 °C, the supernatant was isolated. The remaining liver tissue from each sample was frozen in liquid nitrogen for later transcriptome analysis. To further study the hepatic histology as well as the intestinal microbe taxa alteration induced by high-temperature stress, another three specimens from each tank were randomly selected to be anesthetized. Liver tissues were fixed in 4% paraformaldehyde solution for 36 h, while gut content was collected using sterile Eppendorf tubes and stored in liquid nitrogen immediately for later microbe measurement.

### 2.2. Antioxidant Enzyme Activity Assay

The activities of superoxide dismutase (SOD, JL-T0781), catalase (CAT, JL-T0900), glutathione peroxidase (GSH-Px, JL-T0879), and malondialdehyde (MDA, JL-T0761) were determined according to the protocol recommended by the manufacturer (Jianglai biotechnology, Shanghai, China). Enzyme activities were measured according to the manufacturer’s instructions. Liver samples were homogenized with the cold extraction solution from the detection kits at a ratio of 1:9 (*w*:*v*). After being centrifuged at 14,000 rpm for 10 min at 4 °C, the fresh homogenate was collected into sterile tubes. The WST-8 (water-soluble tetrazolium-1) method was applied to determine the SOD activity at 450 nm [23]. CAT activity was assessed by quantifying the degradation of hydrogen peroxide at 510 nm [24]. GSH-Px activity was determined by the measurement of GSH concentration at 412 nm, as it catalyzes the oxidation of GSH by Cum-OOH [25]. MDA content was calculated based on the reaction of the generated of the generated substrate with thiobarbituric acid at 532 and 600 nm [26].

### 2.3. RNA Extraction, Library Preparation and Transcriptome Sequencing

To compare the differentially expressed genes (DEGs) among different groups, three samples from 2 °C and 22 °C were used for transcriptome analysis. The standard TRIzol reagent (Invitrogen, Carlsbad, CA, USA) was employed to extract total RNA from liver tissue in accordance with the manufacturer’s protocol. RNA quality (purity, concentration) and integrity were assessed using a NanoDrop ND-1000 spectrophotometer (Thermo Scientific, Waltham, MA, USA) and 1% agarose gel electrophoresis, respectively. The qualified samples were used to construct the RNA sequencing library, while mRNA was enriched using oligo (dT) beads and then cleaved into short fragments. Then, first-strand cDNA was synthesized using reverse transcriptase, RNase H, dNTP, and buffer. Following purification and end-repair, paired-ends transcriptome sequencing was performed using an Illumina HiSeq^TM^ 2500 platform. All raw data were analyzed by Allwegene Tech Co., Ltd. (Beijing, China).

### 2.4. Transcriptome Assembly and Differentially Expressed Genes Screening

The mapped reads from each sample were assembled using a reference genome-based approach with the StringTie tool, followed by the calculation of normalized fragments per kilobase of transcript per million values to analyze the gene expression levels. Clean data were analyzed using DESeq2 to recognize the DEGs between the liver tissue of the high-temperature and control groups. The threshold for DEGs’ filtration was q value < 0.005 and |log_2_ (fold change)| ≥ 1. Gene ontology (GO) enrichment and Kyoto Encyclopedia of Genes and Genome (KEGG) pathway enrichment analysis for DEGs were performed based on the hypergeometric distribution using the GOseq package and KOBAS 3.0.

### 2.5. Gut Microbiota Taxa Analysis

After genomic DNA was extracted, 1% agarose gel electrophoresis was used to detect the integrity of DNA samples. Then, the purity and concentrations of nucleic acid samples were verified using a NanoDrop 2000 spectrophotometer (Thermo Scientific Inc., Waltham, MA, USA). Then, the 16S rRNA gene was amplified using universal primers 338F (5′-ACTCCTACGGGAGGCAGCAG-3′) and 806R (5′-GGACTACNNGGGTATCTAAT-3′). Operational taxonomic units (OTUs) were obtained using the Vsearch tool (v2.7.1), after the raw data underwent quality control and chimera filtering. Subsequently, OTU sequences were classified into corresponding species categories using the Bayesian model, and the microbe taxa composition was analyzed at the phylum, family, and genus levels. A Venn diagram was used to identify unique and shared OTUs between the C (2 °C group) and T (22 °C group) groups. Alpha diversity analysis was implemented using the QIME software (v1.8.0) to calculate the Chao1, Shannon, and Simpson indices. Principal coordinate analysis (PCoA) based on weighted Unifrac distance metrics and partial least squares discrimination analysis (PLS-DA) were used to perform beta diversity analysis to examine the structural variance of the gut microbiota among groups. KEGG enrichment annotations were performed using PICRUSt2 to predict the specific microbial functions in burbot under heat stress conditions.

### 2.6. Histopathology Analysis

Five liver tissue samples per group were immediately fixed with 4% paraformaldehyde solution for 36 h. Next, these tissues were dehydrated with different concentrations of alcohol. Then, all liver tissues were sectioned to 3 μm after being encased in paraffin. Finally, tissues were stained with hematoxylin and eosin (HE). The histopathological alterations in the liver were observed using a microscope (Leica, Weztlar, Germany). Cell nuclei were stained blue by hematoxylin, while the cytoplasm was stained pink by eosin.

### 2.7. Immunohistochemistry Detection

Fixed tissues were sliced and then deparaffinized with xylene, followed by rehydration through a graded ethanol series. For antigen retrieval, sections were immersed in citrate buffer (pH 6.0) at 28 °C. Endogenous peroxidase activity was quenched by incubating the sections in 3% H_2_O_2_ for 25 min. After blocking with 3% bovine serum albumin (BSA) for 65 min, the sections were dried in a desiccating oven. Primary antibodies against FAS and Caspase 3 were applied, and the sections were incubated overnight at 4 °C. This was followed by a 70 min incubation with horseradish peroxidase (HRP)-conjugated IgG. The fresh diaminobenzidene (DAB) solution for color development was subsequently applied, after which the sections were counterstained with hematoxylin, dehydrated, and mounted. Sections were scanned using the CaseViewer 2.4.0.119028 (3DHISTECH, Budapest, Hungary), which displayed the nuclei in blue and the positive expression areas of the target proteins in brown.

### 2.8. Statistics Analysis

All results are presented as the mean ± standard deviation (SD), and the data were analyzed using the SPSS statistical software (Version 22.0). The results were checked for normality of distribution (Shapiro–Wilk test) and homogeneity of variance (Levene test). One-way analysis of variance (ANOVA) with Duncan’s test was performed for multiple comparisons between groups. *p* < 0.05 was considered the significance threshold. The Kruskal–Wallis test was applied to analyze bacterial relative abundances, with FDR correction for multiple testing. Pearson correlation analysis was used to determine the relationship between bacterial relative abundance and liver injury. All data were visualized using GraphPad Prism 8.4.3 (686). *p* < 0.05 was considered the significance threshold.

## 3. Results

### 3.1. Effects of Heat Stress on the Mortality of Wild Burbot

The results showed that the higher the temperature, the higher the mortality rate of wild burbot (Table 1). No deaths were recorded at temperatures between 7 and 12 °C. The mortality rate of burbot was 15% at 17 °C, rising to 67% at 22 °C.

### 3.2. Effects of High-Temperature Stress on Oxidative Stress Parameters

**Figure 2:**
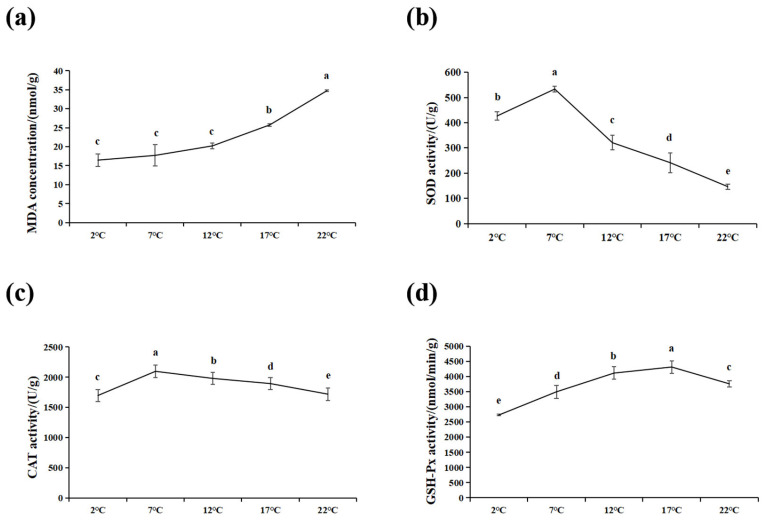
Changes in liver oxidative stress parameters in burbot after heat stress (for n = 3 biological replicates). (**a**) MDA concentration. (**b**) SOD activity. (**c**) CAT activity. (**d**) GSH-Px activity. Different lowercase letters represented significant differences (*p* < 0.05) among different temperature group (means ± SD). One-way ANOVA test was used to identify the statistical significance. MDA: malondialdehyde, SOD: superoxide dismutase, CAT: catalase, GSH-Px: glutathione peroxidase.

The effects of acute heat stress on the hepatic biochemical indices in burbot were clear. The MDA content significantly increased under high-temperature stress (17 °C and 22 °C) compared to the control group (*p* < 0.05) (Figure 2a). SOD, CAT, and GSH-Px activities significantly elevated and then decreased with the increase in breeding temperature (*p* < 0.05) and reached their maximum levels at 7 °C (Figure 2b), 7 °C (Figure 2c), and 17 °C (Figure 2d), respectively. As expected, the SOD and CAT activities at 17 °C and 22 °C were significantly lower than in the control group (*p* < 0.05).

### 3.3. Changes in Gene Expression of Fish Liver

The control (2 °C group) and case groups (22 °C group) obtained a total of 31,709,6772 raw reads. After filtering low-quality sequences, a total of 31,167,5532 clean reads were obtained. Q20 and Q30 values more than 94.00% (Table S1).

**Figure 3:**
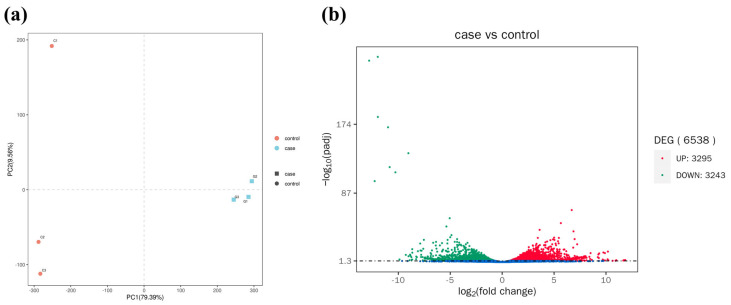
Transcriptomic analyses of the livers after heat stress (for n = 3 biological replicates). (**a**) PCA analysis between control group and 22 °C group. (**b**) Volcano plot for hepatic DEGs. Red and green dots represented significantly up- and down-regulated genes, respectively. Blue dots represented non-significant DEGs.

Principal component analysis (PCA) showed that the samples from the control group (red circle) and those from the case groups (blue square) were clustered together (Figure 3a).

**Figure 4:**
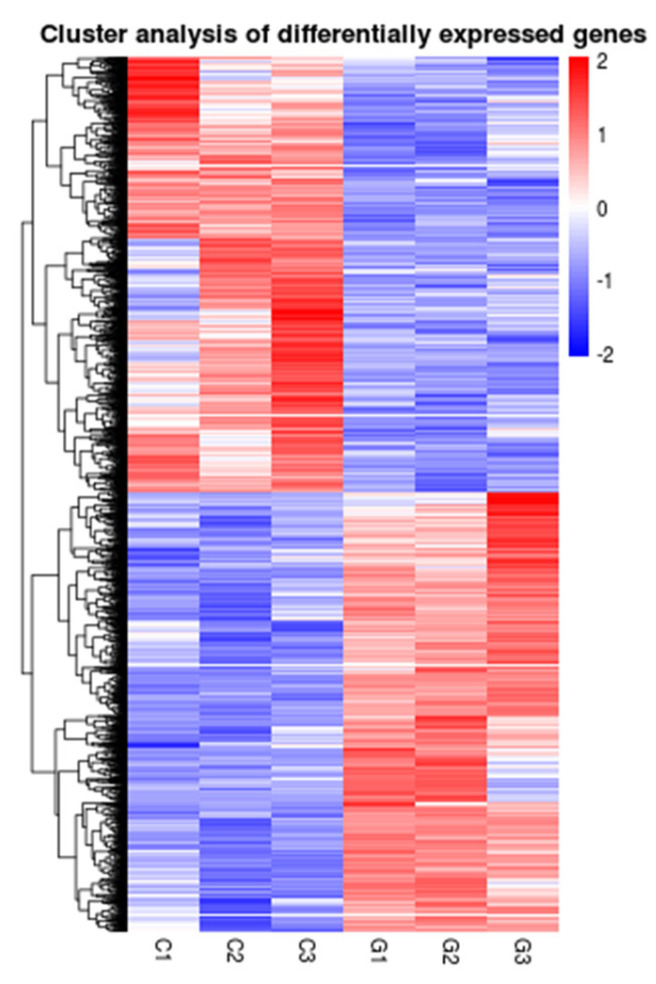
Heatmap of differential genes in livers (for n = 3 biological replicates).

Meanwhile, a total of 6538 DEGs were identified, including 3295 upregulated genes and 3243 downregulated genes (Figure 3b and Figure 4). Many significant energy production (*scd1*, *fabp*, *cpt1*, *hk1*, *hk2*, *alya, aco2*, *idh1*, *ogdh1*, *sdha*), immune (*statla*, *irf9*, *ifnα/β*, *nod1*, *caspase1*, *ripk2*, *tab*, *map3k7*, *mapk*, *tnf-α*, *tlr-2*, *myd88*), and apoptosis (*prf*, *dffb*, *caspase3*, *sptan1*, *atm*, *fas*, *bid*, *ip3r*, *calpain1*, *bax*)-related DEGs were found in the case groups (Table 2).

**Figure 5:**
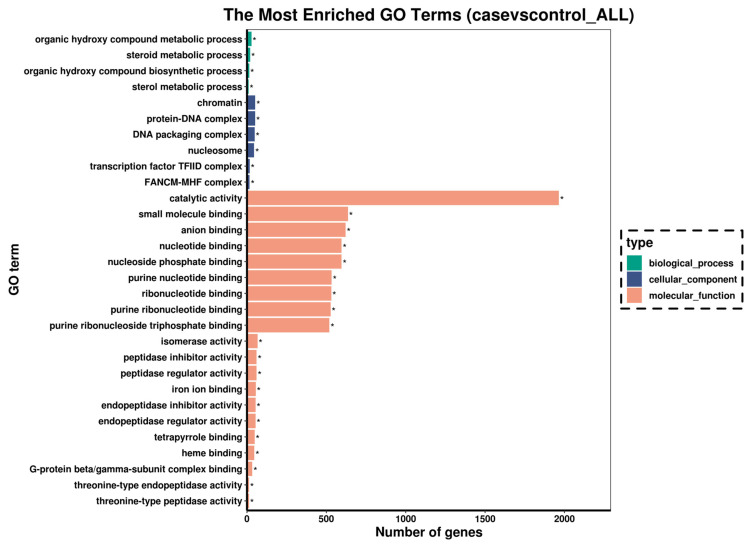
Bar chart of the top 30 significantly enriched of GO categorization of the target genes (for n = 3 biological replicates). Biological processes, cellular components, and molecular functions were represented by green, blue, and orange colors, respectively. “*” represented significantly enriched GO terms.

**Figure 6:**
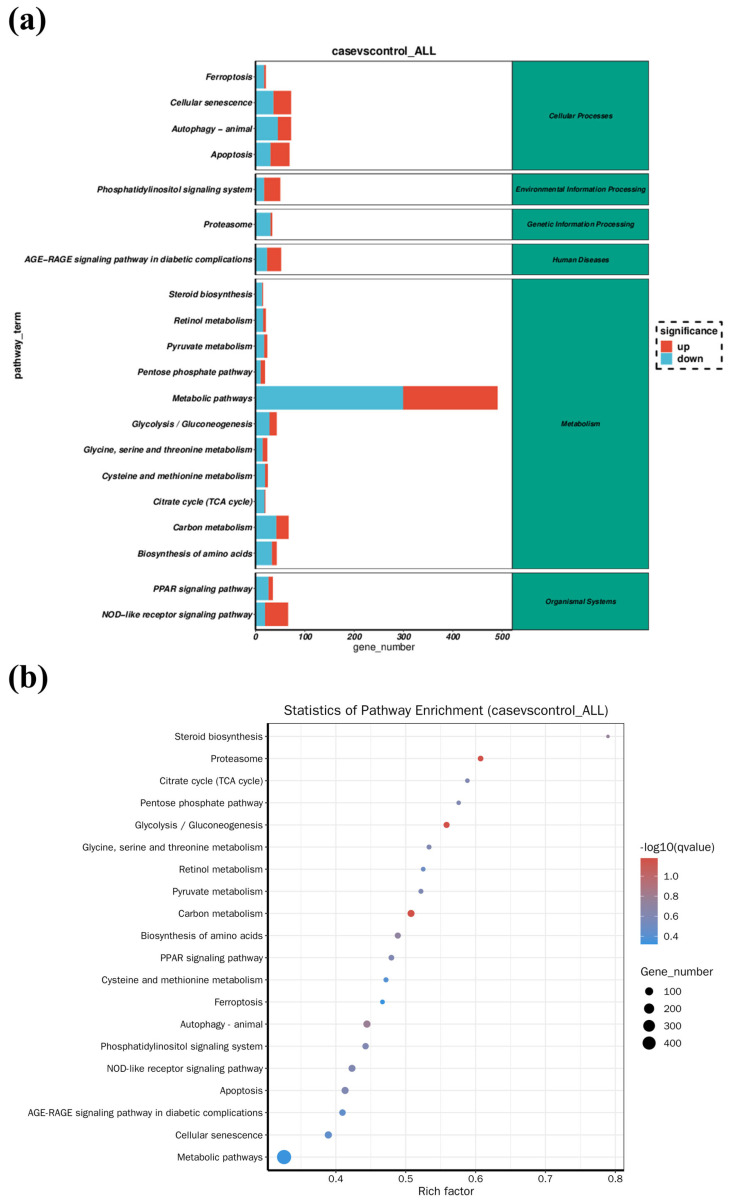
(**a**) Stack diagram for KEGG pathway enrichment analysis (for n = 3 biological replicates). Gene number mapped in each pathways was shown in x-axis while KEGG pathway names were shown in y-axis. Up-regulated and down-regulated genes were represented by red and blue colors, respectively. (**b**) KEGG enrichment bubble plot. The dot size indicated the number of significant DEGs in each pathway while dot color indicated the Q value. The Rich factor is defined as the ratio of the number of differentially expressed genes enriched in the pathway to the number of annotated genes.

To identify the biological functions of the DEGs, GO and KEGG pathway enrichment analysis were implemented. GO annotation for these DEGs was divided into three independent categories including biological process, cellular component, and molecular function. As shown in Figure 5, catalytic activity, small molecule binding, and anion binding were the most representative functions. Further study identified the top 20 significantly different metabolite pathways according to the KEGG analysis (Figure 6a). Among these pathways, a series of signaling pathways associated with carbohydrate metabolism (glycolysis/gluconeogenesis, citrate cycle (TCA cycle), pentose phosphate pathway, and pyruvate metabolism), amino acid metabolism (glycine, serine and threonine metabolism, and cysteine and methionine metabolism), and fatty acid metabolism (PPAR signaling pathway) were significantly enriched. In addition, KEGG enrichment analysis also revealed various DEGs involved in cell death-related signaling pathways (e.g., apoptosis and ferroptosis) and immune response-related pathways (NOD-like receptor signaling pathway and Toll-like receptor signaling pathway) (Figure 6b).

### 3.4. Intestinal Microbe Taxa Analysis

**Figure 7:**
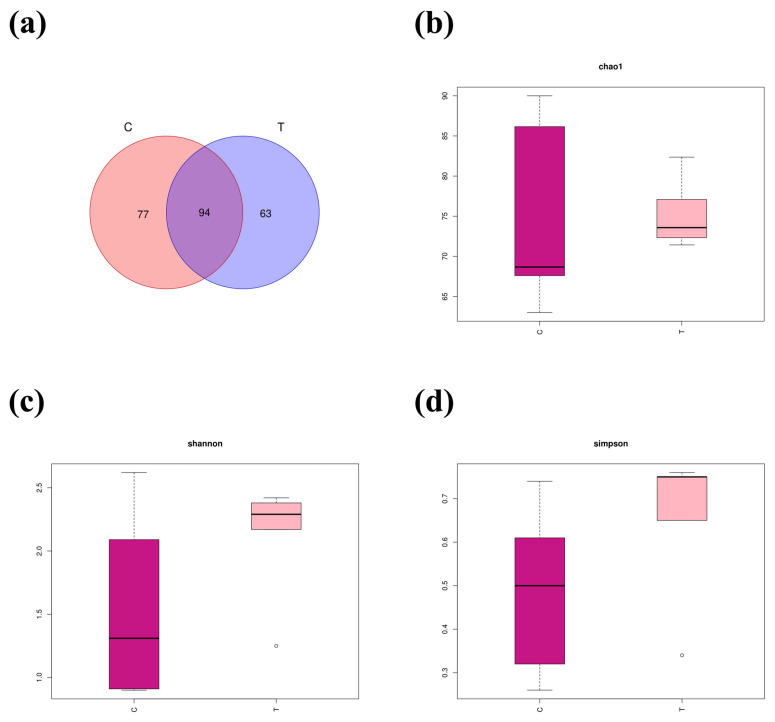
The intestinal microbe changes in *lota lota* under high temperature stress (for n = 5 biological replicates). (**a**) Venn diagram for shared and specific OTUs among two groups. (**b**–**d**) Analysis of alpha diversity of the intestinal microbiota.

**Figure 8:**
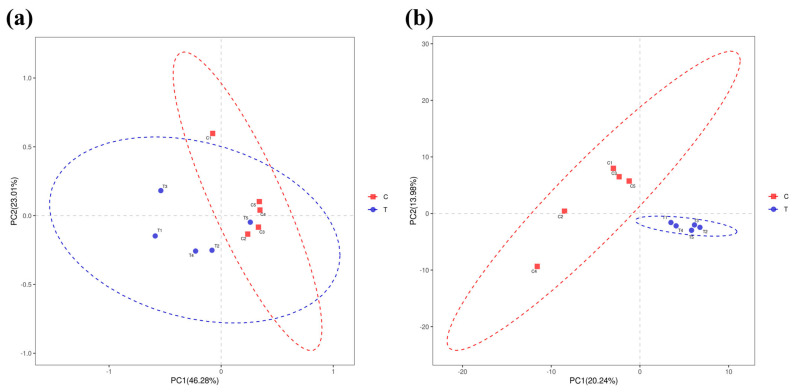
Analysis of beta diversity of the intestinal microbiota (for n = 5 biological replicates). (**a**) Principal coordinate analysis (PCoA) analysis of community difference of two treatment. (**b**) partial least squares discrimination analysis (PLS-DA) analysis of community difference of two treatment.

A total of 811,287 high-quality sequences were obtained from the intestinal microbe samples. A Venn diagram identified a total of 234 OTUs in the C (2 °C) and T (22 °C) groups, with 77 and 63 unique OTUs in the C and T groups, respectively (Figure 7a). Alpha diversity analysis (Figure 7b–d) revealed that the Chao1, Shannon, and Simpson indices between the C and T groups were not statistically significant (*p* > 0.05). PCoA and PLS-DA analysis showed that the composition of the intestinal bacteria flora in burbot individuals exposed to high-temperature condition were obviously different from those in the control group (Figure 8a,b).

**Figure 9:**
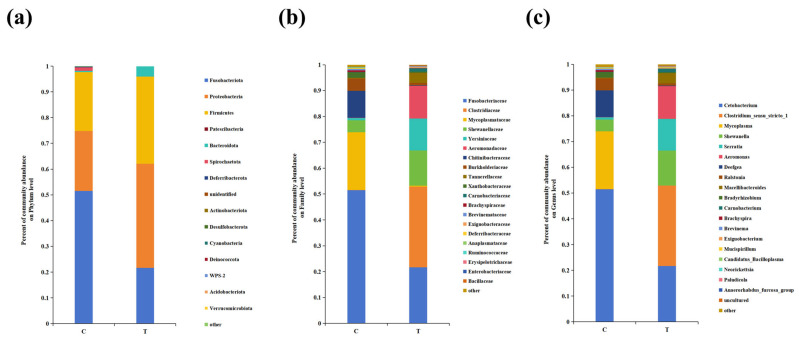
Relative abundances of the gut microbiota at the phylum, family, and genus levels (for n = 5 biological replicates). (**a**) Relative abundances of the gut microbiota at the phylum level. (**b**) Relative abundances of the gut microbiota at the family level. (**c**) Relative abundances of the gut microbiota at the genus level.

**Figure 10:**
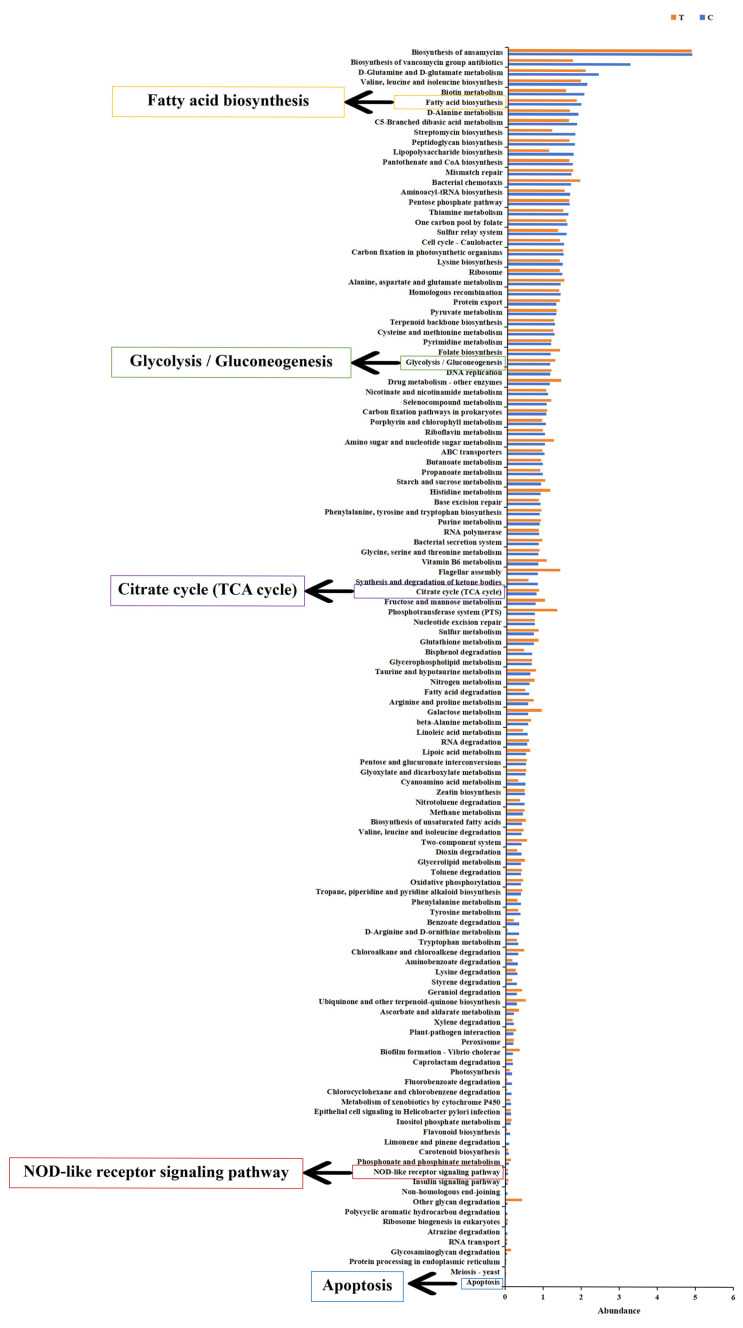
KEGG pathway enrichment analysis under high temperature stress. Data were presented as mean ± SD (for n = 5 biological replicates).

As shown in Figure 9a, at the phylum level, the highest proportion of Fusobacteriota in the control group was 51.5%, followed by Proteobacteria (23.3%) and Firmicutes (23.0%); Proteobacteria dominated in the 22 °C-treatment group, accounting for 40.4%, followed by Firmicutes (33.8%) and Fusobacteriota (21.7%). After 22 °C treatment, the relative abundance of Fusobacteriota significantly decreased at the phylum level, while the relative abundance of Proteobacteria and Firmicutes significantly increased (*p* < 0.05). At the family level, the top three taxa in the control group were Fusobacteriaceae, Mycoplasmataceae, and Chitinibacteraceae, while the top three bacteria flora in the 22 °C-treatment group were Clostridiaceae (31.2%), Fusobacteriaceae (21.7%), and Shewanellaceae (13.6%) (Figure 9b). After 22 °C treatment, the relative abundance of Fusobacteriaceae significantly decreased, while the relative abundance of Clostridiaceae and Shewanellaceae significantly increased (*p* < 0.05). At the genus level, *Cetobacterium* (51.5%) was dominant in the intestinal microbes of the control group, followed by *Mycoplasma* (22.4%) and *Deefgea* (10.4%); *Clostridium_sensu_stricto_1* (31.2%) accounted for the highest proportion of 31.2% in the 22 °C-treatment group, followed by *Cetobacterium* (21.7%) and *Shewanella* (13.6%) (Figure 9c). In addition, the relative abundances of *Cetobacterium*, *Mycoplasma*, *Deefgea*, *Ralstonia*, *Bradyrhizobium*, *Brachyspira*, *Brevinema*, *Mucispirillum*, and *Neorickettsia* decreased (*p* < 0.05) in the 22 °C group compared to the control group, while the relative abundances of *Clostridium_sensu_stricto_1*, *Shewanella*, *Serratia*, *Aeromonas*, *Macellibacteroides*, *Carnobacterium*, *Exiguobacterium*, *Candidatus_Bacilloplasma*, *Paludicola*, and *Anaerorhabdus_furcosa_group* were elevated (*p* < 0.05). In order to better understand how gut microbiota contribute to heat stress adaptation in burbot, we sought to analyze the functional traits of the gut microbial community at high temperature. KEGG function analysis showed that the energy metabolism (fatty acid biosynthesis, glycolysis/gluconeogenesis, and citrate cycle (TCA cycle)), immune function (NOD-like receptor signaling pathway), and cell death-related signaling pathway (apoptosis) were significantly enriched (Figure 10).

### 3.5. Correlation Analysis Between Intestinal Microbes and Liver Antioxidant, Immune, and Apoptosis Indexes

**Figure 11:**
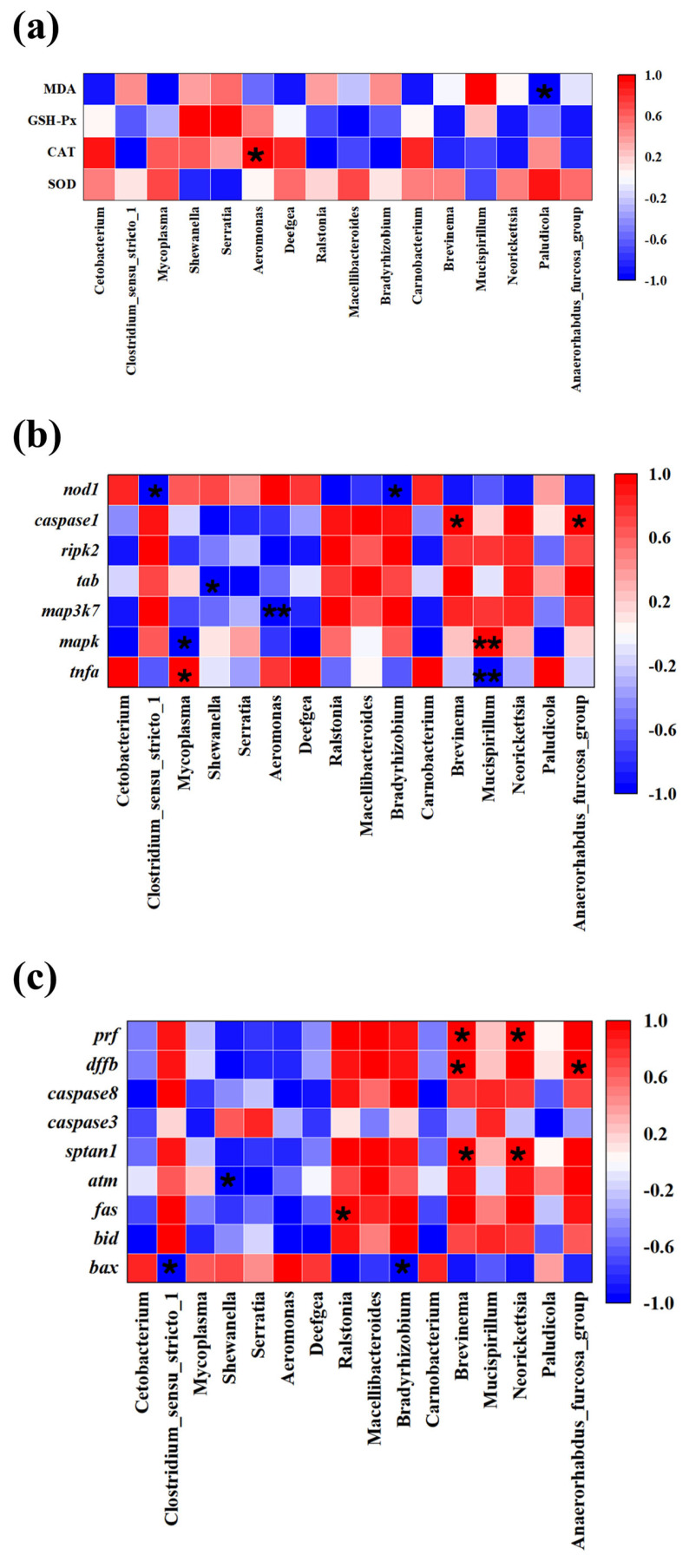
Pearson’s correlation analysis between gut microbiota abundance with hepatic antioxidant indexes, immune/apoptosis genes. (**a**) Correlation analysis between bacterial genera abundance and antioxidant parameters. (**b**) Correlation analysis between bacterial genera abundance and immune genes. (**c**) Correlation analysis between bacterial genera abundance and apoptosis genes. Three biological replicates per group were used. * Represents signicantly negative or positive correlations (* *p* < 0.05; ** *p* < 0.01). Red represents positive correlation while blue color indicates the negative correlation.

Significant correlations were observed between hepatic antioxidant indicators (CAT and MDA) and the gut microbiota (*Aeromonas* and *Paludicola*) (Figure 11a). *Aeromonas* was positively correlated with the CAT level in liver (*R* > 0.5, *p* < 0.05), while *Paludicola* was negatively correlated with the MDA level (*R* < −0.5, *p <* 0.05). Meanwhile, Pearson’s correlation analysis was performed for the gut microbiota and immune function-related genes (Figure 11b). The results from the genus level analysis showed that the change in the relative abundance of *Mycoplasma*, *Brevinema*, *Mucispirillum*, and *Anaerorhabdus_furcosa_group* was significantly positively associated with hepatic *tnfa*, *caspase1*, and *mapk*. However, the relative abundance of *Clostridium_sensu_stricto_1*, *Mycoplasma*, *Shewanella*, *Aeromonas*, *Bradyrhizobium*, and *Mucispirillum* was significantly negatively correlated with *nod1*, *mapk*, *tab*, *map3k7*, and *tnfa*. In addition, the correlations between the gut microbiota and apoptosis related were explored using Pearson’s correlation analysis (Figure 11c). The relative abundances of *Ralstonia*, *Brevinema*, *Neorickettsia*, and *Anaerorhabdus_furcosa_group* were significantly positively correlated with apoptosis-related DEGs (*fas*, *sptan1*, *dffb*, and *prf*), while *Clostridium_sensu_stricto_1*, *Shewanella*, and *Bradyrhizobium* had a significantly negative correlation with apoptosis regulation genes (such as *bax* and *atm*).

### 3.6. Hepatic Histology Observation

**Figure 12:**
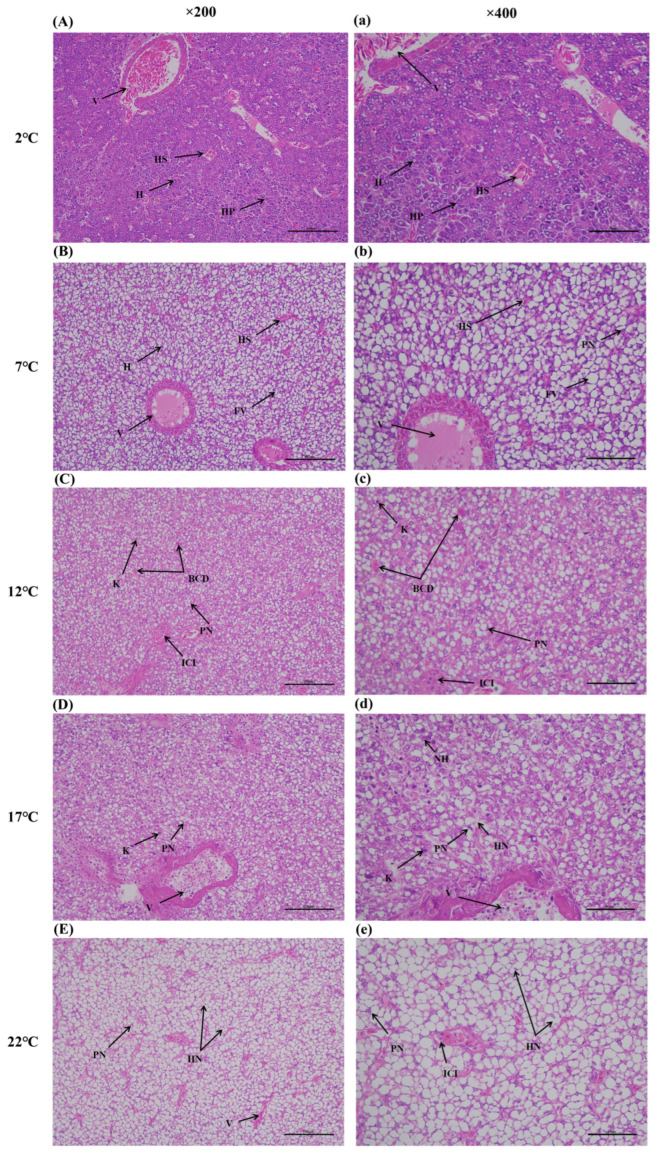
Hepatic histology in lota lota after heat stress. (**A**–**E**) represents liver tissues in the 2 °C (control group), 7 °C, 12 °C, 17 °C and 22 °C group, respectively (×200, scale bar = 100 μm). (**a**–**e**) represents liver tissues in the 2 °C (control group), 7 °C, 12 °C, 17 °C and 22 °C group, respectively (×400, scale bar = 50 μm). V: vein; H: hepatocytes; HS: hepatic sinuses; HP: hepatic plate; FV: fat vacuoles; PN: cellular peripheral nucleus; ICI: Inflammatory cell infiltration; BCD: blood cell deposition; K: karyolysis; NH: nuclear hypertrophy; HN: hepatocyte necrosis.

In order to confirm whether heat stress would induce hepatic injury, pathological observation results were determined, as shown in Figure 12. The liver tissue in the control group showed a normal shape, the hepatocytes exhibited obvious boundaries, and nuclei were regularly distributed in the center of the cells (Figure 12a). The hepatic plate in the 7 °C groups was distributed chaotically, and fat vacuoles and cellular peripheral nuclei were observed (Figure 12b). After 12 °C exposure for 96 h, inflammatory cell infiltration, blood cell deposition, cellular peripheral nuclei, and karyolysis were observed in the liver tissues (Figure 12c). The liver issues in the 17 °C groups showed nuclear hypertrophy, cellular peripheral nuclei, karyolysis, and hepatocyte necrosis (Figure 12d). Liver tissue in the high-temperature group (22 °C) displayed plentiful hepatocyte necrosis, fat vacuoles, and cellular peripheral nuclei (Figure 12e).

### 3.7. Effect of Heat Stress on FAS and Caspase3 Expression in Burbot

**Figure 13:**
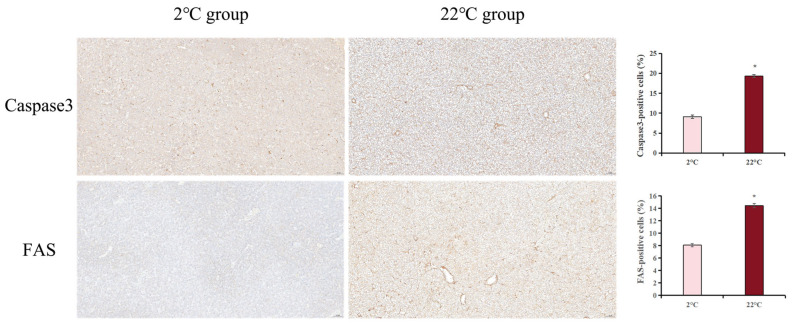
Immunohistochemical staining for the apoptosis-related proteins in burbot liver tissues (400×). Scale bar, 50 μm (for n = 3 biological replicates). Quantifications of IHC staining are shown in the panel right. The nucleus was blue and the positive expression area of the target protein was brown. * above the bars represents significant differences (*p* < 0.05) between groups.

Immunohistochemistry was used to analyze the levels of apoptosis-associated proteins (factor-related apoptosis (FAS) and cysteinyl aspartate specific proteinase 3 (Caspase3)) (Figure 13). In the liver, significantly higher FAS and Caspase3 levels were found in the 22 °C group when compared with the control group (*p* < 0.05).

## 4. Discussion

Global warming could contribute to increasing water temperatures and the occurrence of extreme heatwave events, presenting a challenge for freshwater fish [27]. The *lota lota* population usually completes their breeding migration and spawning behavior in 1.9–3.3 °C, and the roe incubation temperature range is also extremely narrow (1.8–3.0 °C) [7]. Therefore, *lota lota* is particularly sensitive to increasing water temperatures and could be regarded as an ideal animal model to explore the physiological response mechanism in aquatic organisms under thermal stress conditions.

MDA is a byproduct of lipid peroxidation, and its level could indirectly reflect the degree of oxidative stress injury [28]. In this study, there was no significant change in MDA contents between 2 and 12 °C, indicating reactive oxygen species (ROS) were eliminated in a timely manner; hence, the fish maintained normal physiological metabolism. Therefore, there may not be oxidative stress injury between 2 and 12 °C, although the spawning process and embryonic development would be disturbed. Meanwhile, the significantly increased MDA content from 12 to 22 °C suggests that high-temperature stress disrupts the balance in the antioxidant defense system and induces oxidative stress injury in liver. Burbot may be able to activate an autologous physiological defense response to maintain the dynamic ROS balance in liver, when only the reproduction function is disrupted under heat stress conditions. However, once the temperature exceeds the tolerance threshold (22 °C), excessive ROS attack the cell membranes, causing oxidative stress-mediated tissue injury in the liver, which promotes MDA production to accelerate the lipid peroxidation reaction in hepatocytes. Similar results were found for lenok trout (*Brachymystax Lenok tsinlingensis*) under high-temperature stress [29].

Previous studies have reported that temperature changes in the external environment can promote ROS formation, which can prompt the activation of the enzymatic antioxidant defense system in order to prevent oxidative damage [30]. SOD can weaken the negative effects of ROS by changing superoxide radicals into H_2_O_2_. Subsequently, H_2_O_2_ is decomposed into non-toxic H_2_O and O_2_ under the catalysis of CAT and GSH-Px [31]. In this study, SOD and CAT activity in burbot liver increased between 2 and 7 °C, which would enable clearing excessive ROS accumulation in the liver and would not induce cellular lipid peroxidation injury. However, with the increase in the water temperature, the SOD and CAT activities decreased. This suggests that high-temperature stress can cause the antioxidant system to collapse, accelerating oxidative stress injury by inhibiting antioxidant enzyme activities. Taken together, the results suggest that burbot could enhance the antioxidant response to relieve oxidative stress injury by elevating the activities of antioxidase under 7 °C; thus, this temperature represents the heat stress temperature during spawning period. Then, burbot would lose the antioxidant defense capacity once the external stress intensity exceeds its resistance threshold, which would induce oxidative stress injury due to excessive oxygen radical accumulation.

It is noteworthy that substantial fat vacuoles and alterations in tissue structure occurred in female individuals exposed to 7 °C, although this temperature is regarded as normal in summer. It is possible that the optimal survival temperature has a large seasonal shift between the spawning period and non-reproductive periods [32]; females might require a cold environment to ensure normal embryo development in winter. Thus, the seasonal context should be taken into account in all studies investigating the response of *lota lota* to temperature change. However, this hypothesis needs further study. The phenomena of oxidative stress damage, tissue inflammatory response, and cell death were first observed in burbot when the temperature increased to 12 °C. Hence, 12 °C is a “tipping point for normal physiological metabolism status” for burbot, and this temperature may be the critical point at which burbot enter the “damage” phase. Subsequently, liver sections from the 17 °C treatment showed structural damage, and the onset of mortality was observed. It is hypothesized that 22 °C may represent the thermal limit for burbot during the spawning period, at which the mortality rate of the fish reached 67%. The molecular mechanism of the dynamic effects of the thermal limit for the burbot during the reproductive period needs to be further explored. Minimal increases in winter temperatures can induce oxidative stress in females, resulting in the depletion of substantial liver reserves, and the spawning of burbot cannot be induced [7]. These findings provide evidence that winter-specialist species are already seen as vulnerable to temperature increases during winter [33,34]. Maintaining water temperatures below 7 °C is critical for female burbot reproduction and survival.

In order to adapt to dynamic fluctuations in water temperature in a natural habitat, fish can change their metabolism strategies under stress [35]. HK is one key enzyme in the first stage of the glycolysis process. Insufficient ATP supplement in fish under high temperature (22 °C) might reduce the concentration of glucose, leading to the subsequent decreasing trend in HK activity. Previous studies have shown that extremely high temperature conditions can suppress glycometabolism in fish [36]. In the present study, genes (*alya*, *aco2*, *idh1*, *ogdh1*) involved in the TCA cycle were downregulated, which may lead to an interruption in the energy supply in burbot [37]. Meanwhile, genes associated with oxidative phosphorylation, including *ndufc1*, *sdha*, and *ppa1*, showed reduced expression, which may impair electron flow in the electron transport chain. Excessive active electrons could directly react with molecular oxygen to generate ROS, accelerating the cell damage process [38]. PPAR is one main nuclear receptor involved in fat catabolism. In this study, a series of DEGs (*scd-1*, *fabp*, and *cpt-1*) were found to be involved in the PPAR signaling pathway between 2 °C and 22 °C. The fatty acid binding protein (*fabp*) plays important roles in fatty acid transport and peroxisomal β-oxidation [39,40]. *Cpt-1* is located on the inner side of the mitochondrial outer membrane and catalyzes the conversion process of acyl-CoA to fatty acyl [41]; it is the first component and rate-limiting step of the carnitine palmitoyl transferase system [42]. In the biosynthesis of monounsaturated fatty acids, stearoyl-CoA (*scd*) is the rate-limiting enzyme [43]. In the present study, the relative expression of these metabolism-related genes was significantly downregulated at 22 °C, which was similar to the results in Yangtze sturgeon (*Acipenser dabryanus*) [44]. They may regulate their own metabolism mode through energy metabolism pathways under heat stress conditions, resulting in glycometabolism and lipid metabolism disorder.

In fish, the immune system plays a key role in health maintenance and is particularly vulnerable to changes in the external environment [45]. Studies have shown that heat stress can affect the immune response process in aquatic animals [46]. In this study, some immune-related signaling pathways including the NOD-like receptor signaling pathway and Toll-like receptor signaling pathway were significantly enriched. In the NOD-like receptor signaling pathway, many genes (*stat*, *irf9*, *ifnα/β* and *caspase1*) were upregulated in liver under the 22 °C water temperature. The signal transducer and activator family (*stat*) is the essential executor of the cellular antiviral response and adaptive immunity [47]. Interferon regulatory 9 (*Irf9*) can combine with *stat* to form IFN-Stimulated Gene Factor 3 (ISGF3) complex, which is critical for interferon (IFN) gene expression [48]. *Caspase1*, an important inflammatory protease, is responsible for the secretion of pro-inflammatory cytokines such as interleukin (IL)-1β, IL-18, and IL-33 [49]. When the immune system of burbot resists heat stress, the innate immune response might be activated by promoting the expression of the *stat*, *irf9*, ifnα/β, and *caspase1* genes. A thermal stress experiment in black rockfish (*Sebastes schlegelii*) also found that the NOD signaling pathway was involved in the physiological regulation process of initiating the immune defense response [50]. Toll-like receptors (TLRs), a class of pattern recognition receptors associated with innate immunity, initiate cytokine expression upon activation, thereby modulating the development of inflammation [51]. *TLR2*, which has been shown to be the principal mediator for macrophage activation, can recognize diverse bacterial products and activate B cells to produce antibodies and synthesize pro-inflammatory molecule *TNF-α* via the Phosphatidylinositol 3-kinase (PI3K)-protein kinase B (AKT) signaling pathway [52,53]. In our study, the significant upregulation of *tlr2*, *akt*, and *tnf-α* in liver suggests that heat stress promotes the B lymphocyte to secrete a series of pro-inflammation media in burbot liver through the crosstalk among various signaling molecules.

Apoptosis is a programmed cell death pathway, which plays an important role in cell development and host immune modulation [54]. Death receptor-mediated apoptosis can maintain normal physiological homeostasis. Factor-related apoptosis ligand (*fasl*) binds to transmembrane receptor (*fas*) and then recruits Fas-associated death domain (FADD), which results in the formation of the death-inducing signal complex (DISC), activating the apoptosis executor *caspase3* [55]. Meanwhile, inositol 1,4,5-trisphosphate receptor type 3 (*ip3r*) mediates cell apoptosis by activating the expression of *calpain 1* [56]. In the present study, the expression of *fas*, *ip3r*, *calpain1,* and *caspase3* showed an increased trend under a 22 °C water temperature, indicating that acute heat stress accelerates apoptosis, eliminating the damaged hepatocytes caused by high-temperature exposure. In addition, our study demonstrated that FAS and Caspase3 protein expression levels were markedly elevated in liver after high-temperature exposure. Taken together, burbot could initiate this compensatory mechanism (the spontaneous cell death pathway) to resist the negative effects of heat stress on their metabolism and maintain homeostasis.

The intestinal microbe community alters when a host faces external environmental stress [57]. It has been reported that a warmer climate significantly affects the relative abundance of dominant bacteria species [58]. In this study, the main phylum in the control group was Fusobacteriota, while Proteobacteria was the dominant phylum in the 22 °C group. Previous research has shown that Fusobacteriota is involved in the immune regulation of fish and might play an anti-inflammatory role in the intestinal tract by producing butyrate [59]. There was an evident decline in Fusobacteriota abundance under heat stress. Hence, high temperature might affect Fusobacteriota colonization, inhibiting the secretion of butyrate, which would indirectly disrupt the host immunity and disease resistance of burbot. Proteobacteria is a dominant phylum in many fish species, such as Chinook salmon (*Oncorhynchus tshawytscha*) and rainbow trout (*Oncorhynchus mykiss*) [3,60]. Xavier et al. [61] showed that Proteobacteria are involved in intestinal inflammatory development, and the change in Proteobacteria abundance has been considered a signature of intestinal microbial disorder. Based on this, the elevated trend in Proteobacteria could be regarded as a biomarker for the intestinal microenvironment disorder caused by heat stress. In addition, increases in the abundances of Firmicutes and Bacteroidota phylum were also found in *lota lota* under high-temperature stress. According to one study, intestinal Firmicutes and Bacteroidota play essential roles in the production of short-chain fatty acids, carbohydrate fermentation, and polysaccharide catabolism utilization in fish [62]. Our study suggests that burbot accelerate the energy metabolism process to meet the physiological requirements under heat stress by promoting the colonization of Firmicutes and Bacteroidota. In addition, KEGG pathway prediction showed that gut microbes in burbot might be involved in fatty acid biosynthesis, glycolysis/gluconeogenesis, the citrate cycle (TCA cycle), the NOD-like receptor signaling pathway, and apoptosis under thermal stress conditions. In summary, these results indicate that heat stress might mediate programmed cell death, energy metabolism, inflammation development, and immune response in *lota lota* by regulating the gut microbe community composition.

An intestinal microbe imbalance is connected with metabolism disorder in animals. The gut affects the liver through the absorption of endotoxins, microbial metabolites, and nutrients [19]. The liver releases bile acids and numerous bioactive substances into the intestinal tract through bile ducts and systemic blood flow [63]. *Aeromonas* is a fish pathogen, which can promote the secretion of endotoxin, which induced liver injury in zebrafish [64]. In this study, *Aeromonas* was positively correlated with the expression of antioxidant enzyme activities. It was found that the relative abundance of the *Aeromonas* genus increased under heat stress, as did the enzyme activities of these antioxidant indicators. Organisms could enhance their antioxidant response to resist the inflammatory injury caused by the excessive colonization of *Aeromonas*. Furthermore, correlation analysis showed that the observed change in the *Anaerorhabdus_furcosa_group* was positively correlated with the changes in the hepatic immune/apoptosis-related genes in the high-temperature group, indicating that the immune/apoptosis response could be stimulated by promoting the colonization of the intestinal *Anaerorhabdus_furcosa_group*. *Anaerorhabdus_furcosa_group* is a beneficial microbiota in *Bacteroidetes* phylum, which can produce large levels of acetic acid to mediate the anti-inflammatory immune response [65]. *Bacteroidetes* is closely associated with the promotion of intestinal inflammation and apoptosis of intestinal cells [66]. Consistent with the previous literature [67], the *Anaerorhabdus_furcosa_group* genus could promote intestinal inflammation development and the cell apoptosis process. However, the detailed molecular mechanism of the regulatory effects of the *Anaerorhabdus_furcosa_group* genus on inflammation induction and programmed cell death still needs further research.

## 5. Conclusions

Oxidative stress injury induced by high-temperature stress triggered an immune response and energy metabolism disorder, which eventually led to intestinal and liver damage. Moreover, the thermal limit for burbot induced changes in the structure of the gut microbiota; hence, the gut–liver axis may play a crucial role in this change. The study enhances knowledge regarding how *lota lota*—a cold-water fish—responds to heat stress, revealing potential mechanisms that facilitate its adaptation to warmer environments and providing a theoretical basis for the conservation of *lota lota*.

## Figures and Tables

**Table 1:** The effect of temperature on the mortality of wild burbot.

Temperature Groups	Mortality (%)
2 °C	0%
7 °C	0%
12 °C	0%
17 °C	15%
22 °C	67%

**Table 2:** Summary for DEGs in the control and case groups related to energy production, immune, and apoptosis.

Gene Name	Gene Description	log_2_ (Fold Change)	Regulation Trend	KEGG Pathway
*scd1*	stearoyl-CoA desaturase	−6.8926	down	PPAR signaling pathway
*fabp*	fatty acid-binding protein	−3.7339	down	
*cpt-1*	fatty acid-binding protein	−3.8176	down	
*hk1*	hexokinase-1	−2.0128	down	Glycolysis/Gluconeogenesis
*hk2*	hexokinase-2	−2.7132	down	
*alya*	ATP-citrate synthase	−1.4418	down	Citrate cycle (TCA cycle)
*aco2*	aconitate hydratase	−1.5861	down	
*idh1*	isocitrate dehydrogenase	−1.3379	down	
*ogdhl*	2-oxoglutarate dehydrogenase-like	−1.9731	down	
*sdha*	succinate dehydrogenase	−0.93966	down	
*stat1a*	signal transducer and activator of transcription 1a	2.2886	up	NOD-like receptor signaling pathway
*irf9*	interferon regulatory factor 9	1.4744	up	
ifnα/β	interferon alpha/beta	4.7766	up	
*nod1*	nucleotide-binding oligomerization domain-containing protein 1 isoform X1	1.434	up	
*caspase1*	caspase b	2.0607	up	
*ripk2*	receptor-interacting serine/threonine-protein kinase 2	−1.9997	down	
*tab*	TAK1-binding protein	−1.2793	down	
*map3k7*	mitogen-activated protein kinase kinase kinase 7	3.2473	up	
*mapk*	itogen-activated protein kinase	1.6597	up	
*tnf-α*	tumor necrosis factor a	3.298	up	Toll-like receptor signaling pathway
*tlr2*	toll-like receptor 2	2.3241	up	
*myd88*	myeloid differentiation primary response protein MyD88	1.3693	up	
*akt*	RAC-gamma serine/threonine-protein kinase	1.9379	up	
*prf*	perforin	2.3632	up	Apoptosis
*dffb*	DNA fragmentation factor subunit beta	−1.6189	down	
*caspase8*	caspase 8	−1.3553	down	
*caspase3*	caspase 3	1.3808	up	
*sptan1*	spectrin alpha chain, non-erythrocytic 1	0.98599	up	
*atm*	serine-protein kinase ATM	2.0906	up	
*fas*	tumor necrosis factor receptor superfamily member 6	1.5171	up	
*bid*	BH3 interacting domain death agonist	−1.2813	down	
*ip3r*	inositol 1,4,5-triphosphate receptor type 1	2.2898	up	
*calpain1*	calpain-1 catalytic subunit	1.1952	up	
*bax*	apoptosis regulator BAX	−1.2334	down	

## Data Availability

The data that support the findings of this study are available from the corresponding author upon reasonable request.

## Supplementary Materials

The following supporting information can be downloaded at: https://www.mdpi.com/article/10.3390/antiox14080947/s1, Table S1: Quality control of the RNA-seq data obtained from different samples.
