# Supplementary material for: Combined Impacts of Acute Heat Stress on the Histology, Antioxidant Activity, Immunity, and Intestinal Microbiota of Wild Female Burbot (Lota Lota) in Winter: New Insights into Heat Sensitivity in Extremely Hardy Fish
Source: Antioxidants (Basel). 2025 Jul 31;14(8):947. doi: 10.3390/antiox14080947 (PMC12382668; doi:10.3390/antiox14080947)
Supplement: Supplementary file 1 [file antioxidants-14-00947-s001.zip › Supplementary materials.pdf]

Table S1. Quality control of the RNA-seq data obtained from different samples.

Sample	Raw Reads	Raw Bases	Clean Reads	Clean Bases	Q20	Q30	GC Content
C1	58314344	8.74G	57404156	8.48G	97.69%	94.01%	46.98%
C2	55840790	8.37G	54711956	8.03G	97.95%	94.61%	47.97%
C3	51200438	7.68G	50219620	7.37G	97.86%	94.41%	48.18%
G1	53875578	8.08G	52915208	7.82G	97.80%	94.18%	46.96%
G2	50630694	7.59G	49791210	7.36G	97.72%	94.01%	46.61%
G3	47234928	7.08G	46633382	6.88G	97.85%	94.34%	46.59%
